# The Anti-Inflammatory Effects of CXCR5 in the Mice Retina following Ischemia-Reperfusion Injury

**DOI:** 10.1155/2019/3487607

**Published:** 2019-07-04

**Authors:** Xing Cao, Wen Li, Ying Liu, Hu Huang, Chang-Hua Ye

**Affiliations:** ^1^Aier School of Ophthalmology, Central South University, Aier Eye Institute, Changsha, Hunan, China; ^2^Department of Ophthalmology, Changsha Aier Eye Hospital, Changsha, Hunan, China; ^3^Department of Ophthalmology, Hunan Children' Hospital, Changsha, Hunan, China; ^4^Mason Eye Institute, Department of Ophthalmology, School of Medicine, University of Missouri, Columbia, MO 65212, USA

## Abstract

**Object:**

Retinal ischemia-reperfusion (I/R) injury is a common pathological process in many ophthalmic diseases; there are no effective therapeutic approaches available currently. Increasing evidence indicates that microglia mediated neuroinflammation plays an important role in the retinal I/R injury. In this study, we aimed to investigate the roles of chemokine receptor CXCR5 in the pathological process of retinal I/R injury model.

**Method:**

Retinal I/R injury model was established in CXCR5 knockout and wild mice by the acute elevation of intraocular pressure (AOH) for 60 minutes, and the eyes were harvested for further analyses. The cellular location of CXCR5 was detected by immunofluorescence staining; the expressions of CXCR5 and CXCL13 after I/R injury were analyzed by quantitative RT-PCR. The retinal microglia were detected as stained for Iba1 (+). Leakage of inflammatory cells was observed on the H&E stained cryosections. The protein expression and quantification of zonula occludens (ZO-1) were determined by Western blotting and densitometry. Capillary degeneration was identified on the intact retinal vasculatures prepared by trypsin digestion.

**Results:**

The number of activated microglia marked by Iba1 antibody in the retina was increased after retinal I/R injury in both KO and WT mice, more significant in KO mice. The leakage of inflammatory cells was observed largely at 2 days after injury, but there was no or little leakage at 7 days. The number of inflammatory cells (mainly neutrophils) was greater in CXCR5 KO mice than in WT mice, mainly located under internal limiting membrane. CXCR5 deficiency led to more ZO-1 degradation in CXCR5 KO mice compared to C57BL6 WT mice 2 days after reperfusion. The cellular capillaries were also significantly increased in the KO mice compared to the WT mice.

**Conclusion:**

Our findings suggest that the chemokine receptor CXCR5 may protect retina from ischemia-reperfusion injury by its anti-inflammatory effects. Thus, CXCR5 may be a promising therapeutic target for the treatment of retinal I/R injury.

## 1. Introduction

Retinal ischemia-reperfusion (I/R) injury is a common pathological process in many ophthalmic diseases, including retinal vascular occlusion, glaucoma, and diabetic retinopathy [1, 2]. There is no cure for these diseases and the available treatments are not very effective currently. The I/R damage is a dual process, in which timely restoration of blood flow after ischemia is very critical to reduce neuronal apoptosis; however, it also results in local and systemic inflammatory responses and aggravates oxidative stress and inflammatory damage [3]. A better understanding of the molecular mechanisms involved in retinal I/R injury is very important for the development of new therapies to minimize and even reverse vision loss. Increasing researches suggest that neuroinflammation plays a pivotal role in the pathogenesis of retinal I/R injury, which is typical of inflammatory responses induced by microglial proinflammatory activation [4–7]. Microglia activation is associated with increased cytokine expression and oxidative stress, which in turn leads to further activation of microglia as well as the infiltration of circulating immune cells like macrophage [8].

Chemokines are chemotactic cytokines that control the migration and localization of all immune cells. Chemokines function by binding to the corresponding chemokine receptors. There are about 20-30 chemokine receptors in mammalian genome including humans. These chemokine factors were grouped into four classes based on their cognate ligands (C, Cc, Cxc, and Cx3c) [9]. Chemokine receptors are hypothesized to play roles in the pathogenesis of glaucoma, because they can regulate the migration of immune and inflammatory cells. The chemokine receptor CX3CR1 regulates microglial neurotoxicity in an experimental mouse glaucoma model; CX3CR1 deficiency increased microglial neurotoxicity and subsequently induced more extensive RGC loss than control mice with CX3CR1 [10]. A high concentration (1000 ng) of MCP-1/CCL2 exacerbated RGC loss in an experimental glaucoma model; 100 ng MCP-1/CCL2 provided neuroprotection towards RGC. Neuroprotective MCP-1/CCL2 (100 ng) also upregulated the immunoreactivity of insulin-like growth factor-1 (IGF-1) in the RGCs [11]. CXCR5 is a member of the Cxc subfamily [9]; it is expressed constitutively or induced by various cell types, such as inflammatory cells, RPE, and neuronal progenitors. B-cells and T-cells expressing CXCR5 can be attracted to the inflammatory sites by CXCL13, its corresponding ligand [12, 13]. CXCR5 has been linked to a wide range of diseases such as neuropathic pain, breast cancer, arthritis, HIV, and chronic graft-versus-host disease [14–17]. In brain ischemia stroke model, some researchers found that infarct volume and proinflammatory M1 microglia/macrophage density are increased in CXCR5 knockout mice, suggesting that microglia-derived CXCL13 acting through CXCR5 might be involved in neuroprotection following stroke through recruiting B lymphocytes to the ischemic hemisphere and secreting IL-10 [18, 19]. Roles of CXCR5 have also been shown in some researches related to eye diseases or conditions. For example, in a spontaneous autoimmune mouse model of uveitis (R161H), it is discovered that well-organized lymphoid aggregates in the retina and it has tertiary lymphoid tissue (TLT) characteristics. The chemokine receptor CXCR5 and its ligand CXCL13 were upregulated notably, and eyes with lymphoid aggregates showed lower inflammatory scores via fundus examination and a slower initial rate of loss of visual function by electroretinography, compared with eyes without these structures [20]. The expression of CXCR5 is upregulated in cultured microglia in the activated state by LPS [21]. CXCR5 is a protective factor of retinal cells in aged mice and its loss leads to pathological changes similar to age-related macular degeneration (AMD) [22]. These findings indicate that CXCR5 may be involved in the regulation of neuroinflammation in ocular tissues as part of CNS, mainly associated with lymphocytes and microglia. However, the role of CXCR5 in retinal I/R injury remains unknown.

The acute elevation of intraocular pressure (AOH) induced injury is a well-established model used to research the mechanisms of and potential therapy for retinal ischemia-reperfusion injury [23–25]. In this study, we demonstrate that acute high intraocular pressure could cause neuroinflammation and injure retina; the absence of CXCR5 exacerbates this inflammatory response and retinal damage. Our findings suggest that CXCR5 may have a role in the pathogenesis of retinal I/R injury.

## 2. Materials and Methods

### 2.1. Animals

The CXCR5^−/−^ (KO) mice [B6.129S2 (Cg)-CXCR5tm1Lipp/J] and wild-type (C57BL/6J) mice were obtained from Jackson Laboratory. Animals were housed on a 12-hour light-dark cycle animal facilities, which are pathogen-free. The mice were fed with normal chow diets and provided with water ad libitum. All experimental procedures and protocols in this study were approved by the Animal Care and Use Committee of Central South University and performed in accordance with the Association for Research in Vision and Ophthalmology (ARVO) guidelines. In both C57 WT and CXCR5 KO groups, 5-10 mice were used for H&E and immunofluorescence staining; 5 mice were used for Western blot and quantitative RT-PCR analysis and 10 mice for preparation of complete retinal vascular bed.

### 2.2. Retinal Ischemia-Reperfusion (I/R) Injury Model

The mice were anesthetized with a mixture of ketamine hydrochloride (50mg/kg), xylazine (10mg/kg), and acepromazine (2mg/kg) by intraperitoneal injection. The cornea was anesthetized with topical 0.5% proparacaine hydrochloride, and the pupil was dilated with 1% tropicamide. The anterior chamber of the right eye was cannulated with a 30G infusion needle connected to an aseptic normal saline reservoir, which was elevated slowly to the height of 120cm to maintain an intraocular pressure of 80-90 mmHg for 60 min, which was confirmed by pale retina under microscope. TONOLAB tonometer (Icare Finland Oy, Vantaa, Finland) was used to measure the intraocular pressure by the same people at the same time. The contralateral eye was used as control and cannulated with a 30G needle connected to normal saline just for puncturing anterior chamber. After the procedure, 0.3% tobramycin ointment was applied to the conjunctival sac. The animals were allowed to recover for 2d or 7d before sacrifice unless otherwise specified.

### 2.3. H&E Staining

Mice were sacrificed at scheduled time point. The eyes were enucleated. The cornea, lens, iris, and ciliary body were removed, and the remaining eyecups were immersed in 4% paraformaldehyde overnight. The eyecups were then dehydrated in 15% sucrose, followed by 30% sucrose, and embedded in OCT (Tissue-Tek O.C.T. Compound 4583, USA). The OCT specimens were cryosectioned into 10 microns thickness. The sections were immersed in 4% paraformaldehyde for 5min and then incubated in Harris hematoxylin for 2min followed by rinse in PBS for 3 times and then stained with 0.5% eosin Y solution for 20sec. The staining was visualized and imaged with microscope; the leakage of retinal inflammatory cells was counted under microscope on each section.

### 2.4. Immunofluorescence Staining

The cryostat sections were obtained as mentioned above. They were blocked and permeabilized with 10% goat serum and 0.25% TritonX-100 in PBS for 1h. Primary rabbit anti-ionized calcium binding adaptor molecule-1 (Iba-1) antibody was used for microglia (1:150, Abcam, UK), and anti-CXCR5 antibody was used for CXCR5 (1:100, Abcam, UK) overnight at 4°C and then washed in PBS 3 times for 5 min each. Secondary antibody conjugated to either Alexa 488 (1:1000) or Cy3 (1:300) was applied for 1.5h at room temperature, used to visualize the staining under fluorescence microscope (Axio-pan2; Carl Zeiss Meditec, Inc.), and Zeiss Axion 4 software was used for collecting immunofluorescence staining information. The total number of Iba1-positive cells in the retina sections was quantified.

### 2.5. Quantitative RT-PCR

Total RNA was extracted from retina 2 days after reperfusion by using a commercial kit (Omega Bio-tek, Inc. Georgia, USA)and then reverse-transcribed to cDNA; cDNA was synthesized (Vazyme Biotech Co., Ltd., Nanjing, China) in a final reaction volume of 20 *μ*l. Real-time PCR was performed using ChamQ Universal SYBR qPCR Master Mix reagent (Vazyme Biotech Co., Ltd., Nanjing, China) with a qPCR system (CFX Connect Real-Time PCR Detection System, Bio-Rad, Hercules, CA, USA), and the following primers were used: CXCR5, forward: 5′-CATGGGCTCCATCACATACA-3′, reverse: 5′-GTGCCTCTCCAGGATTACCA-3′; CXCL13, forward: 5′-TCTGGAAGCCCATTACACAA-3′, reverse: 5′-AGCTTGGGGAGTTGAAGACA-3′; GAPDH, forward: 5′- ACTTTGTCAAGCTCATTTCC-3′, reverse: 5′- TGCAGCGAACTTTATTGATG-3′. The relative quantification of gene expression was analyzed by the 2^-ΔΔCq^ value.

### 2.6. Western Blot Analysis

Mice were killed 2 days after modeling. The eyes were enucleated and excised annularly along limbus. The cornea, lens, iris, and ciliary body were removed. The retina was detached, separated, and lysed in lysis buffer [150mM Sodium chloride, 20mM tris (pH 7.4), 2mM EDTA, 1%Triton X-100, and EDTA protease inhibitor]. The retinal specimen was further homogenized with ultrasound at 4 degrees for 3-5 sec and incubated for 30 min. The retinal lysis was centrifuged at 14000 rpm for 10 min at 4°C. Supernatant was collected and protein concentration was determined by BCA protein concentration detection. About 20-30ug protein was subjected to SDS-PAGE and then transferred onto a nitrocellulose membrane and blocked in 5% fat-free milk for 1h. It was incubated overnight at 4°C with the primary antibody of ZO-1(1:500, DHSB) and then incubated with the secondary antibody which was combined with horse radish peroxidase (HRP). The band densities, normalized to the *β*-actin band, were analyzed with ImageJ software. The ZO-1 protein expression levels in different groups were normalized to the CXCR5 KO-injured group, which sets up as 1-fold. Each Western blot analysis was repeated 3 times with 5 samples in each group.

### 2.7. Complete Retinal Vascular Bed and Analysis of Capillary Degeneration

Mice were killed 7 days after the establishment of mouse model. The eyes were enucleated and immersed in 10% formalin immediately for 24h at room temperature. Retina was dipped in 10% formalin immediately for 24h at room temperature again and then transferred and rinsed overnight with deionized water. Retina was incubated for 2h at 37°C in the mixed liquor (PH=6.5) containing elastinase (40u/ml, Sigma, USA), phosphate buffer (100mmol/L), sodium chloride (150mmol/L), and EDTA (5.0mmol/L, Sigma, USA) and then transferred to Tris-HCl (100mmol/L, pH=8.5) for 1h at room temperature. Retina was removed of nonvascular tissue with a fine single hair brush and dried at a slide for 10min. The retinal vessels were stained with hyperoiodate-Schiff and hematoxylin as follows: the retina was incubated in hyperiodate solution for 8min, rinsed with distilled water for 10min, and incubated in Schiff's reagent (Sigma, USA) for 10min until the blood vessel began to appear red, which usually occurred in 3-5 min, and then the organization was incubated in hematoxylin solution (Harris Hematoxylin, Sigma, USA) for 15min at room temperature, flush with distilled water and check blood vessel color, dipped retina in 0.05% ammonium hydroxide solution 20 times. The retina was dipped in alcohol with different concentration gradients, 50% alcohol solution for 10 sec, 95% alcohol solution for 30 sec, and 100% alcohol solution for 1 min, and in xylene solutions for 5 times. Pictures were taken on both the middle and peripheral parts of retinal vascular tree, acellular capillaries were defined as capillary-sized vessel tubes without nuclei along their length, and the number per square milliliter of retinal area was quantified and reported.

### 2.8. Statistical Analysis

Statistical analysis was performed with unpaired two-tailed Student's* t*-test using GraphPad Prism 7.03 software. All results were expressed as the mean ± SEM (standard error of the mean). Differences were considered statistically significant when p value is less than 0.05.

## 3. Results

### 3.1. Increased CXCR5 and CXCL13 Expression after AOH-Induced Retinal I/R Injury

**Figure 1:**
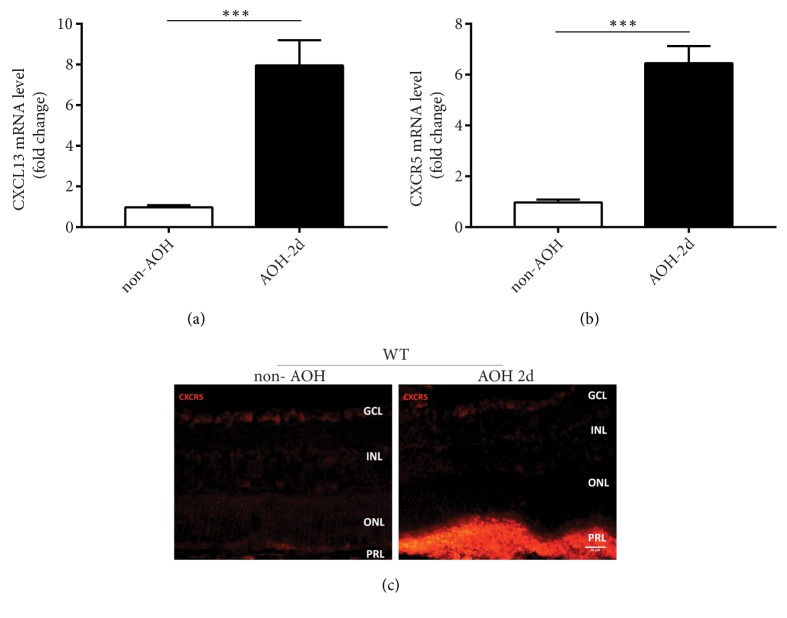
Increased CXCR5 and CXCL13 expression after acute intraocular hypertension- (AOH-) induced retinal I/R injury. (a-b) Quantitative RT-PCR for CXCL13 and CXCR5 mRNA levels in C57WT retina under non-AOH or AOH-2d. (c) Anti-CXCR5 immunostaining of retina sections in C57 WT retina under non-AOH or AOH-2d. Data are means ± SEM. n = 5 animals per group. *∗∗∗P* < 0.001. Scale bar: 30 *μ*m.

To investigate whether CXCR5 signaling pathway is implicated in retina, we examined the expression of CXCR5 and CXCL13, which is the only ligand for CXCR5 in a mouse model of retinal ischemia-reperfusion injury induced by acute elevation of intraocular pressure (AOH). Analysis of mRNA by quantitative RT-PCR showed that CXCL13 (Figure 1(a)) and CXCR5 (Figure 1(b)) expressions were increased in retinal tissue 2 days after reperfusion. Immunolocalization analysis released that the cellular location of CXCR5 was present in the ganglion cell layer (GCL) and outer nuclear layer (ONL) in noninjured retina. After I/R injury, the immunoreactivity for CXCR5 was increased robustly in cells in the photoreceptor layer (PRL) (Figure 1(c)).

### 3.2. The Absence of CXCR5 Aggravates the Activation of Retinal Microglia after I/R Injury

**Figure 2:**
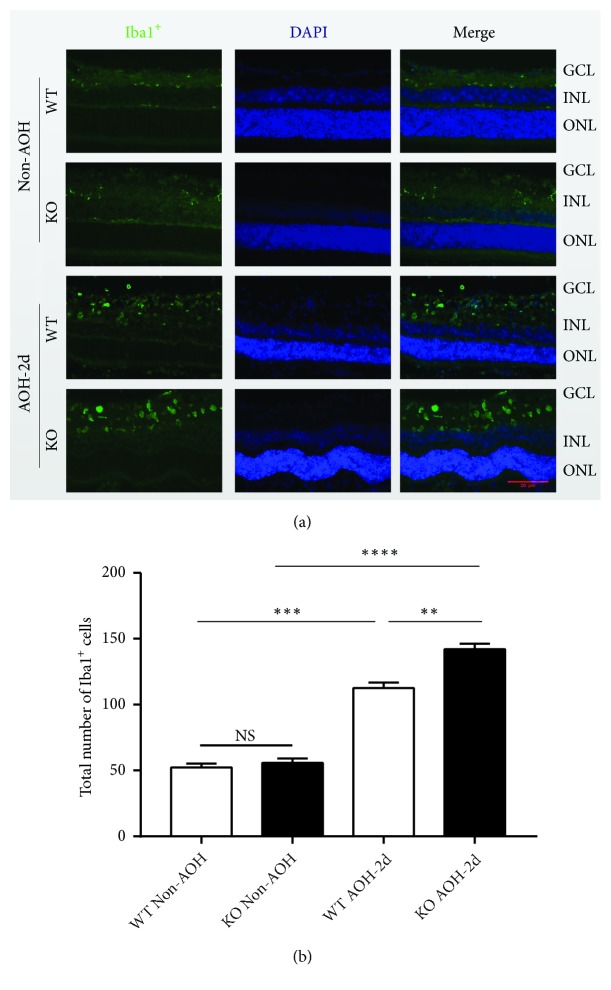
The absence of CXCR5 aggravates the activation of retinal microglia at 2 days after retinal ischemia-reperfusion induced by AOH injury. (a) Anti-Iba1 immunostaining of retina sections in the CXCR5 group and C57 WT group under AOH-2d or non-AOH. (b) Comparison of the total numbers of microglia in the whole retina. Data are means ± SEM. n = 10 animals per group. *∗∗P* < 0.01, *∗∗∗P* < 0.001, and *∗∗∗* *∗P*<0.0001. Scale bar: 20 *μ*m.

To assess the possibility that chemokine receptor CXCR5 may regulate microglia, we stained the retina with an antibody against Iba1, a microglial marker (Figure 2(a)). In the non-AOH-injured retina of C57 WT and CXCR5 KO group, Iba1-positive cells were rarely observed (Figure 2(a) did not show any ramified microglia). But, in the AOH-injured retina, Iba1-positive cells were increased obviously, and microglia had round shapes without visible cell processes, which indicated microglial activation induced by acute intraocular hypertension. In the non-AOH-injured retina, the total number of microglia showed no significant difference between WT and KO groups (*P*=0.2384). In the AOH-2d injured retina, the activated microglia of CXCR5 KO group were significantly more than WT group (*P*=0.0081), and in the CXCR5 KO group, stronger staining signals were also observed, which indicates upregulation of active microglial markers and therefore more active status of microglia and the upregulation of the microglial population by the absence of CXCR5.

### 3.3. The Absence of CXCR5 Aggravates the Accumulation of Retinal Inflammatory Cells in I/R Injury Mice

**Figure 3:**
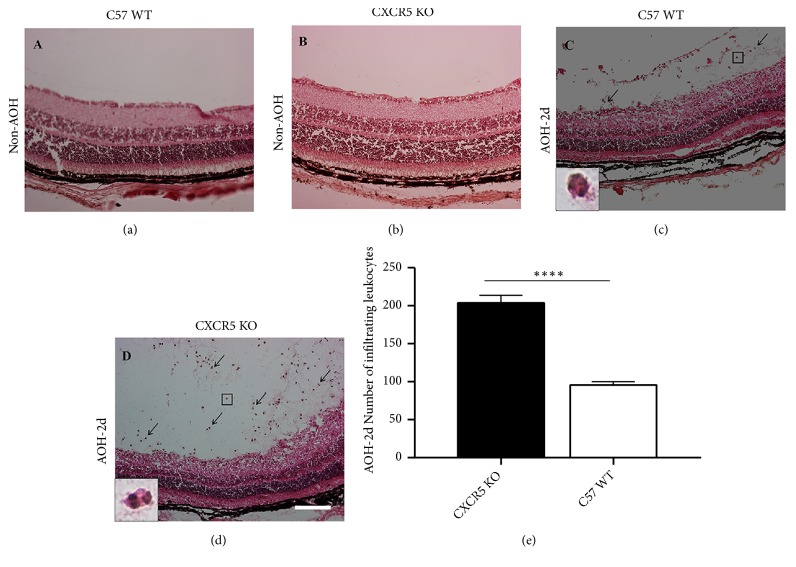
The leakage of inflammatory cell at retina after I/R injury in KO and WT mice. (a)–(d) The leakage of inflammatory cells at retina after AOH injury. Inflammatory cells were indicated by the arrow. (e) The quantitative comparison of inflammatory cell leaking into retina between CXCR5 KO and WT mice following injury. Data are means ± SEM; n = 10 for both groups. *∗∗∗* *∗ P*< 0.0001. Scale bar: 50 *μ*m.

In both WT and CXCR5 KO mice, we found the accumulation of inflammatory cells in retina after acute intraocular hypertension. These inflammatory cells showed multilobed nuclei are mainly neutrophils, probably from peripheral circulation system at two days after acute elevated intraocular pressure is most obvious (Figures 3(a)–3(d)), but at the seventh day there were no infiltrated cells in the vitreous cavity. Seepage cells were located under the internal limiting membrane (ILM). Compared to WT, the seepage cells in CXCR5 KO mice were significantly higher (Figure 3(e)).

### 3.4. The Absence of CXCR5 Aggravates Retinal ZO-1 Degradation in I/R Injury Mice

**Figure 4:**
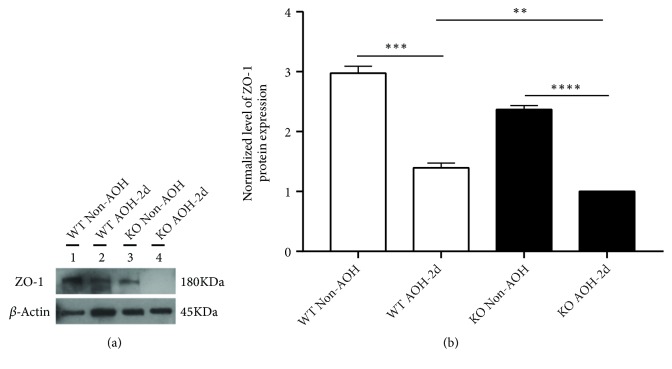
The absence of CXCR5 aggravates retinal ZO-1 degradation in retinal ischemia-reperfusion mice. (a) Western blots (WB) for ZO-1 protein levels in retina. (b) WB quantification of retinal ZO-1 protein level. The normalized optical density ratio of CXCR5 KO group acted as a baseline value. The results were expressed as the mean fold change against the baseline value. Data are means ± SEM. n = 5 animals per group. *∗∗P* < 0.01, *∗∗∗P* < 0.001, and *∗∗∗* *∗P*<0.0001.

The increased infiltration of inflammatory cells following ischemic injury suggests that retinal inner blood retinal barrier (iBRB) was impaired. Therefore, we investigated the effects of acute AOH and CXCR5 absence on the protein expression levels of tight junction protein zonula occludens-1 (ZO-1), an essential component of BRB complex that plays a critical role in maintaining barrier properties. WB results showed that AOH caused a reduction of ZO-1 protein expression levels when comparing AOH with non-AOH control. Cxcr5 absence aggravated the degree of ZO-1 degradation: protein bands were even not visible in the KO mice with AOH for 2 days (Figure 4). Further densitometry analysis of the target protein bands showed that the expression of ZO-1 was significantly higher in uninjured groups than in injured ones in either WT group or KO group (p<0.001).

### 3.5. The Absence of CXCR5 Aggravates the Degeneration of Retinal Capillary Vessel after I/R Injury

**Figure 5:**
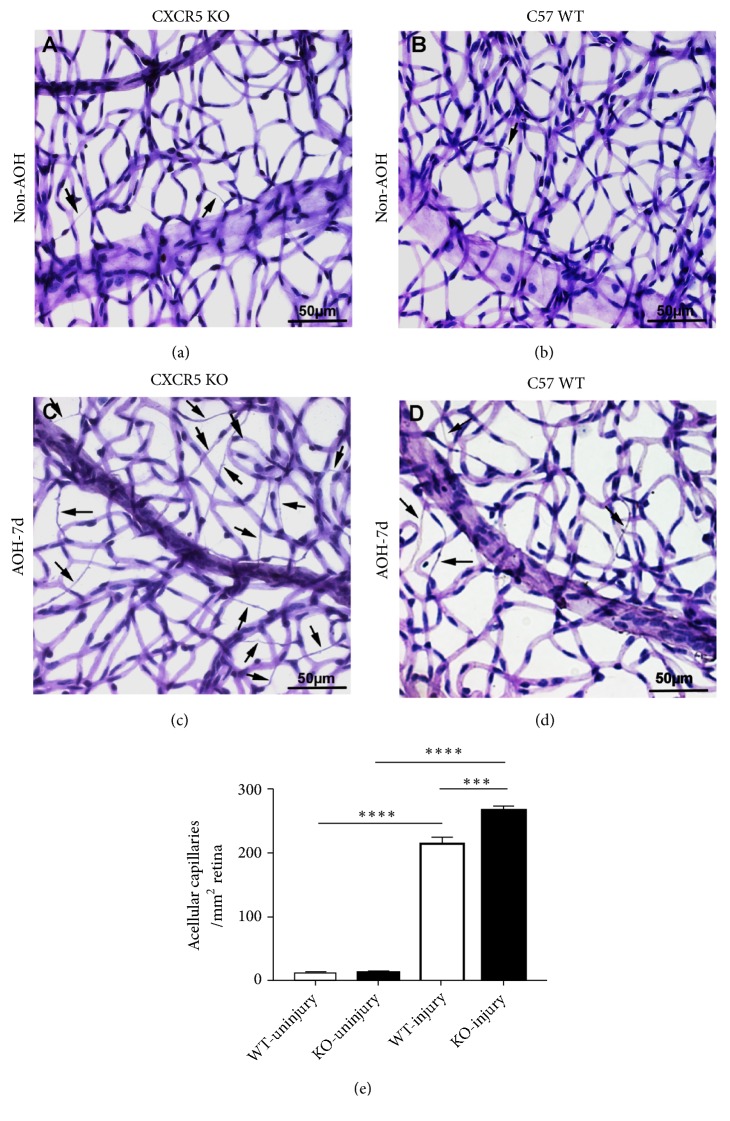
The absence of CXCR5 aggravates the degeneration of retinal capillary vessel after I/R injury. Acellular capillaries were defined as capillary-sized vessel tubes without nuclei along their length, indicated with black arrowheads. (a)–(d) Representative images from respective groups. Acellular capillaries are indicated by arrowheads. (e) Quantification of acellular capillaries. Data are means ± SEM. n = 10 animals per group. *∗∗∗P* < 0.001 and *∗∗∗* *∗P*<0.0001. Scale bar: 50 *μ*m.

In CXCR5 KO mice and WT mice, we found the increase in formation of acellular (degenerate) capillaries in the injured retinas compared to noninjured retinas 7 days after acute high IOP injury. The vascular degeneration in KO group was more obvious compared to WT group (Figure 5); the chemokine CXCR5 showed protective effect on the retinal vasculature.

## 4. Discussion

Although more and more researches have been conducted on the chemokine receptor CXCR5, little attention has been paid to its role in retinal diseases such as ischemia-reperfusion (I/R) injury. In our study, the cellular location of CXCR5 was present in the ganglion cell layer (GCL) and outer nuclear layer (ONL) in noninjured retina. After I/R injury induced by acute ocular hypertension (AOH), CXCR5 was increased obviously in the photoreceptor layer (PRL); the mRNA levels of CXCR5 and its ligand CXCL13 were also increased markedly; these data suggest that the CXCL13/CXCR5 axis may have a role in the pathogenesis of retinal I/R injury. By blocking CXCR5 with gene deletion, we demonstrated the association between CXCR5 and retinal I/R injury ulteriorly. The absence of CXCR5 aggravated the activation of microglia and the accumulation of inflammatory cells in retina after I/R injury. In addition, CXCR5 deficiency led to more retinal zonula occludens-1 (ZO-1) degradation; the retinal vascular degeneration was also increased in the CXCR5 knockout mice with acute ocular hypertension.

Inflammation has been implicated as a secondary injury mechanism following ischemia [26]. Neuroinflammation progresses from hours to days following ischemic stroke and is characterized by the activation of microglia and infiltration of circulating inflammatory cells in cerebrum [27]. In retina, microglial cells become activated after injury. And in activated state these cells have morphological changes, proliferation, and modified expression of enzymes and receptors and release a variety of inflammatory factors, such as NO, tumor necrosis factor (TNF-a), and interleukin (IL-6) [28]. Excessive microglia activation might prompt the release of cytotoxic factors, leading to neuronal damage. It has been observed that activated microglia significantly contributed to the pathophysiology of RGC death [10, 29], and with the treatment of minocycline or a high dose of irradiation, there was a reduction of microglial activation and thus lower RGC death [30, 31]. In this study, the absence of CXCR5 aggravated the activation of retinal microglia after acute intraocular hypertension injury, suggesting that CXCR5 can inhibit the activation of retinal microglia after I/R injury.

In Central Nervous System (CNS), as innate immune cells, microglia closely interact with blood-brain barrier (BBB) screening the infiltrating molecules and, via cytokine/chemokine release, influencing the infiltration of peripheral cells. Excessive activated microglia can directly reduce BBB integrity by promoting the degradation of tight junction protein, increasing endothelial cell permeability [32, 33]. Microglial proinflammatory activation is associated with increased cytokine expression, such as TNF-a, IL-6, and oxidative stress, which in turn leads to the infiltration of circulating immune cells. All of these inflammatory responses are referred to as neuroinflammation [8]. Neuroinflammation can damage critical support cells such as the astrocytes and brain endothelial cells which form the blood-brain barrier (BBB). It is well known that the retina originates from the diencephalon and has many functional and structural features similar to those of the brain. The pathophysiological process of ischemia-reperfusion is similar to that of the brain. In the eye, the endothelial cells of the inner retinal vasculature constitute the inner blood retinal barrier (iBRB), which provides close monitoring of the flow of immunomolecular components between the blood circulation and the retina [34]. Zonula occludens-1 (ZO-1), well known as an essential intracellular component of tight and adherent junctions, plays a critical role in maintaining barrier properties and controlling paracellular permeability of the iBRB, and its degradation will damage the integrity of iBRB. Weakening of the barrier promotes the infiltration of circulating leukocytes and may further aggravate inflammation and retinal damage [35]. In fact, the activation of microglia, increase of inflammatory factors, breakdown of BRB, and infiltration of circulating immune cells were identified in retinal ischemia-reperfusion injury induced by acute ocular hypertension, and some anti-inflammation drugs could prevent retinal neuroinflammation and vascular permeability such as exendin-4, minocycline, and compound chrysin [36–38]. Recruitment and accumulation of circulating immune cells leaking from retinal vessels might be responsible for the increased retinal capillary degeneration [39]. In our study, ZO-1 was more degraded in CXCR5 KO group than in WT group, and the leakage of retinal inflammatory cells (mainly neutrophils) was more prominent in CXCR5 KO group after I/R injury. As a result of acute ocular hypertension, the integrity of iBRB was damaged and the vascular permeability of retina increased, exacerbating the inflammatory cells migration to retina. The retinal capillaries were persistently exposed to the immune-damaged microenvironment and the vascular degeneration became inevitable. We postulated that immune inflammation and vascular degeneration interact to aggravate retinal I/R damage caused by acute high intraocular pressure.

Despite the findings in the current study, there still exist limitations regarding the roles of chemokine receptor in the pathogenesis of retinal ischemia-reperfusion. First, this study was only focused on neuroinflammation and vascular damage after retinal ischemia-reperfusion. We did not investigate the potential ganglion cell damage. Second, recent studies on microglial cell have increasingly tended to its phenotypes, neurotoxic proinflammatory M1 activation, and neuroprotective anti-inflammatory M2 activation. More explicit studies would be done to address whether CXCR5 gene is involved in the regulation of microglial M1/M2 polarization and how these phenomena are implicated in the pathogenesis of retinal I/R injury.

In conclusion, our findings demonstrated that the chemokine receptor CXCR5 may prevent neuroinflammation via inhibiting the activation of microglia cells and maintaining the integrity of the iBRB barrier in retinal I/R model induced by acute ocular hypertension. Chemokine receptor CXCR5 may be a new target to prevent retina from damage following retinal I/R injury. Further studies are needed to address the roles of CXCR5 in the pathogenesis of retinal I/R injury in humans.

## Data Availability

The data used to support the findings of this study are included within the article.
